# Effect of hemodynamics on clot formation and flow cessation in patient-specific coronary bifurcations

**DOI:** 10.1016/j.bpr.2026.100248

**Published:** 2026-01-06

**Authors:** Mohammad Rezaei, Bahar Firoozabadi

**Affiliations:** 1 School of Mechanical Engineering, Sharif University of Technology, Tehran, Iran

## Abstract

Hemostasis prevents bleeding by forming clots, although disruptions can cause insufficient or excessive clotting. Shear stress is a critical factor influencing the normal function of the blood and can stimulate the formation of unnecessary clots. Stenosed vessels significantly contribute to this phenomenon by increasing shear stress on the vessel walls. This study aims to quantify the effects of shear stress and stenosis severity on thrombus formation in patient-specific left coronary bifurcations. Three-dimensional patient-specific geometries were reconstructed from angiographic data. Blood flow was modeled using the Brinkman equation, while transport and interactions of coagulation factors were simulated through the convection-diffusion-reaction equation. Model predictions were validated using literature data. The findings showed that all patients experienced significant shear stress at the stenosed regions, which are highly susceptible to clot formation. Shear stress was found to be inversely related to vessel diameter and directly related to the stenosis degree. Furthermore, in bifurcated vessels, blood flow may reach zero before complete occlusion by the clot, emphasizing the role of hydrodynamic resistance in redirecting blood flow. Among these factors, the stenosis degree emerges as the most significant predictor of blood flow cessation time. A relationship was developed allowing physicians to accurately and rapidly monitor a patient’s condition using angiographic images, providing new insights into the hemodynamic mechanisms of coronary thrombosis and supporting improved diagnosis and treatment of cardiovascular diseases.

## Why it matters

Blood clots within coronary arteries can block blood flow and lead to heart attacks. Understanding where and why these clots grow is essential for early diagnosis and treatment. In this study, we use patient-specific arterial shapes reconstructed from angiographic images to simulate blood flow and clot formation. Our results show that the severity of the stenosis strongly influences how much flow is reduced and where a clot is likely to form. This approach may help clinicians rapidly evaluate clot risk using routine imaging, providing insights into the mechanical factors that cause coronary thrombosis and support more personalized treatment strategies.

## Introduction

The World Health Organization states that cardiovascular diseases are the leading cause of death worldwide (1,2). Therefore, monitoring and treating cardiovascular diseases is a vital necessity (3). Hemostasis, the natural response of the body to prevent bleeding, occurs through vasoconstriction, platelet aggregation, and blood clot formation (4). The primary mechanism of hemostasis is clot formation, which can result from changes in the vessel wall, blood components, or blood flow (Virchow triad (5)). Any disturbance in these factors can lead to thrombosis, which blocks blood vessels and may cause stroke or heart attack (6,7). The innermost layer of the vessel wall is the endothelial cell layer, which is in direct contact with the blood. When endothelial cells are damaged, platelets adhere to exposed collagen, release ADP, and attract more platelets to form a growing clot (8). If the damage to the vessel wall is large, the platelet mass cannot seal the wound, and blood clot formation becomes necessary. In such cases, the coagulation cascade is activated, leading to the production of thrombin (T), which converts fibrinogen (FG) to fibrin (F) to stabilize the clot (7,9). Severe damage to the vessel wall causes clotting within 15–20 s, while minor damage takes 1–2 min (4).

More than 50 chemical agents influence coagulation, including procoagulants that promote it and anticoagulants that inhibit it. In the bloodstream, anticoagulants predominate, and prevent coagulation, but damage to the vessel wall activates procoagulants, leading to clotting. Coagulation happens in three stages: activation of prothrombin (PT) activator, conversion of PT to T, and FG to F fibers via extrinsic and intrinsic pathways (10). A clot changes the vessel geometry, altering blood flow. Shear stress of blood flow can induce damage on endothelial cells of the vessel, contributing to clot formation according to Virchow’s triad (5). Clot formation in bifurcated arteries is clinically important due to complex flow and shear stress gradients, which increase the risk of thrombosis and related events such as stroke and myocardial infarction. High shear stress increases the production of prostacyclin and nitric oxide and inhibits platelet activation, while low shear stress decreases their production, promoting platelet aggregation and plaque formation (11,12,13).

Blood vessels, particularly at the bifurcation of vessels, are exposed to a wide range of shear stresses that differentially regulate atherosclerosis and thrombus formation. In low shear regions, lipoprotein infiltration and endothelial activation promote inflammation and atherosclerotic plaque development (12,14). In contrast, physiological high shear stress is generally athero-protective, maintaining endothelial quiescence, enhancing nitric oxide production, and suppressing adhesion molecule expression (15,16). However, pathologically elevated shear stress and steep shear gradients, such as those occurring in severe stenoses, have a different biological effect: they promote von Willebrand factor unfolding and rapid platelet adhesion, thereby triggering platelet-rich thrombus formation (12,13,17,18). Thus, while low shear contributes to the initiation and progression of atherosclerotic, severe pathological high shear stress facilitates thrombus formation by exposing subendothelial collagen and activating shear-dependent platelet adhesion mechanisms. Several studies have investigated the hemodynamic factors affecting clot formation in stenosed arteries. For instance, Kabir et al. (19) examined the effects of double stenoses with varying degrees of area reduction and demonstrated that increasing stenosis degree significantly alters velocity, pressure, and wall shear stress. Similarly, Yazdani et al. (20) demonstrated that high shear rate plays a significant role in the initiation of thrombosis within stenotic microchannels. Furthermore, Hosseinzadegan and Tafti (1,21) studied clot growth in stenosed tubes and found that platelet adhesion depends on Reynolds number, wall shear rate, and stenosis degree, and, based on their numerical results and calibrated by the experimental data, they developed a relationship between the mentioned parameters.

Simulating blood clot formation is complex due to the multiple reactions involved (22). Several computational approaches have been developed to model thrombus formation, each capturing different aspects of its physiology. Discrete or particle-based methods (23,24,25,26,27), such as the solid-fluid interaction phase-field framework of Zheng et al. (24), provide detailed descriptions of clot mechanics, while representing biochemical aspects of coagulation in a simplified manner. Multiscale platelet-resolved models (28,29), including the approach of Shankar et al. (28), track individual platelets using hybrid neural network-lattice Boltzmann method schemes and provide detailed microscopic insight, but are computationally feasible only at microscale domains. In contrast, continuum biochemical models (30,31,32,33,34) describe the transport, activation, and deposition of species through convection-diffusion-reaction (CDR) equations, providing an appropriate level of biochemical detail while remaining suitable for larger vascular domains.

The continuum method, which varies in complexity based on the blood components, solves Navier-Stokes equations for blood flow and CDR equations for species (30,31,32,33,34). All of these models assume rigid vessel walls and massless blood components. The simplest model, by Lobanov and Starozhilova (32), includes three species: activator, inhibitor, and F. The most complex, the LF model (31), includes 56 species. Intermediate models also exist (30,34,35,36). Complex models are computationally expensive and limited to two-dimensional (2D) microscale geometries, whereas simpler models can be applied to 3D geometries.

A common model, the Sorensen model (34), includes seven species: resting platelets (RPs), activated platelets (AP), adenosine diphosphate (ADP), thromboxane (TX), PT, T, and antithrombin (AT). The shear rate influences diffusion, and consequently the deposition of platelets at the sites of injury. This formulation was later extended by Wu et al. (37), who built upon the Sorensen model (34) by adding clot growth through the inclusion of deposited APs and incorporating a fluid-thrombus interaction term that allows the growing clot to influence the local flow field. This model has been further developed in other studies (1,21,34,37,38,39). In this model, the effect of clot growth on the flow field has not been considered (34). On the other hand, some studies have considered the presence of clots on blood flow (30,32,37,38,40,41,42), while others have not (1,33,34,35,36). Besides, various models have been proposed to describe this alteration as mentioned earlier. Hemodynamic equations also influence the CDR equations directly through flow velocity and indirectly by altering the diffusion coefficient (1,21,34,38,39,40,41) and platelet deposition rate (1). Any disruption in the coagulation triad (vessel wall, blood flow, and blood components) can lead to unnecessary clotting (5). The normal shear stress range in vessels has been reported to be up to 7 Pa and when it exceeds this, the likelihood of thrombosis increases (12).

Since the simulation of blood clot formation is a complex process, only a few studies have investigated clot formation in 3D and, to the best of our knowledge, no studies have been conducted on patient-specific left coronary bifurcation. As highlighted in the recent review by Grande Gutiérrez et al. (43), most thrombosis simulations are performed in simple geometries, while patient-specific multiphysics models remain scarce, particularly for coronary bifurcations. The review also notes that the few patient-specific studies typically lack detailed biochemical clot growth modeling. The studied geometries include: horizontal 3D tubes (30,38), abdominal aortic aneurysms (an abnormal bulge or ballooning in the wall of a blood vessel) (44,45), infarcted left ventricles (33), and left atrial (42,46), and a recent work has modeled clot growth in simple 2D bifurcated vessels based on geometric occlusion and shear rate dynamics, without accounting for biochemical coagulation pathways or platelet activation mechanisms (47).

Most previous models used simplified or 2D geometries, limiting their relevance to real patient anatomy, especially in complex 3D coronary bifurcations. To select the geometry to model the clot formation, it is observed that the complexity of the chosen model of chemical reactions is inversely related to the complexity of the geometry. Consequently, by choosing a complex 3D geometry, such as a bifurcation in the coronary arteries, it is not possible to use highly detailed models of the coagulation cascade.

This study focuses on blood clot formation in patient-specific 3D geometries of the left coronary bifurcation. At first, for validation, the geometry of several published studies is reconstructed, which only includes the first two coagulation mechanisms. The model is then extended to incorporate the third mechanism of clot formation: the conversion of FG to F. Next, to determine the effect of the clot on the flow field, the growth of deposited platelets is added. In this step, the Brinkman equation is used to accurately calculate the effect of clot growth on the flow field. The developed model is then compared and validated with published numerical and experimental results. Finally, the model is applied to the patient-specific 3D geometries of the left coronary bifurcation. This study models the post-endothelial injury state in the stenosed left coronary bifurcation. Areas of high shear stress are identified as high-risk areas and considered as sites of initial injury. This study combines patient-specific 3D geometries with hemodynamic and biochemical modeling to simulate clot growth and flow cessation in coronary bifurcations.

## Materials and methods

In this study, blood is modeled as an incompressible Newtonian fluid. This assumption is justified because blood behaves approximately Newtonian at shear rates above 100 s^–1^ (48,49), and the shear rates in our stenotic coronary simulations consistently exceed this range. Furthermore, recent findings show that using a Newtonian model has a negligible impact on platelet transport and deposition in high shear stenotic flows (50).

In this work, the effect of clot growth on the flow field is also incorporated by modeling the clot as a porous medium. To achieve this, the Brinkman equation (Eq. 2) is applied as the governing equation over the entire computational domain, considering that this equation has also been used in previous studies (31,40,41) to simulate dynamic clot growth. However, the permeability coefficient (Brinkman coefficient) is defined as a function of the concentration of F and deposited platelets (40). As a result, in clot-free regions, where the concentration of F and deposited platelets is zero, the last term of the Brinkman equation becomes negligible, effectively reducing the equation to the Navier-Stokes equation.

The continuity (51) and Brinkman equations (52) are presented below.

(1) $$\nabla \cdot \vec{V}=0$$

(2) $$\rho \frac{d\vec{V}}{dt}+\rho \left(\vec{V}\cdot \nabla \right)\vec{V}=-\nabla p+\nabla \left(\mu \nabla \cdot \vec{V}\right)-\frac{\mu }{G_{clot}}\vec{V}$$

In the above equations, *ρ* represents the density, *p* denotes the pressure field, $\vec{V}$ indicates the blood flow velocity, and *μ* refers to the dynamic viscosity of the blood.

### Effect of clot growth

The term $-\frac{\mu }{G_{clot}}$ in Eq. 2 represents the Brinkman coefficient. *G*_*clot*_ is the permeability coefficient of the porous medium, and $\frac{1}{G_{clot}}$ corresponds to its hydraulic resistance (40). In this study, it is assumed that the permeability coefficient depends on the concentration of F and deposited platelets, which are the primary components of a clot (53). Consequently, the total hydraulic resistance of the blood clot, which is attributed to deposited platelets and F, is assumed to be (40):

(3) $$\frac{1}{G_{clot}}=\frac{1}{G_{DP}}+\frac{1}{G_{F}}$$

*G*_*DP*_ and *G*_*F*_ represent the permeability associated with deposited platelets and F, respectively. The contribution of deposited platelets to the total hydraulic resistance is described as follows (40):

(4) $$\frac{1}{G_{DP}}=\frac{1}{{G_{DP}}_{\max}}\left(\frac{{\left(\varphi \right)}^{2}}{{\left(\varphi_{Cri}\right)}^{2}+{\left(\varphi \right)}^{2}}\right)$$

$\frac{1}{{G_{DP}}_{\max}}$ represents the maximum hydraulic resistance due to platelet deposition. *φ* denotes the ratio of deposited platelets to the maximum possible platelet deposition, and *φ*_*Cri*_ is the threshold ratio of deposited platelets. When *φ* = *φ*_*Cri*_, the hydraulic resistance reaches half of its maximum value. In this study, the Davis equation (54) is employed to predict the hydraulic resistance of F:

(5) $$\frac{1}{G_{F}}=\frac{16\psi^{1.5}\left(1+56\psi^{3}\right)}{r_{F}^{2}}$$

where *r*_*F*_ represents the radius of the F fibers, and *ψ* denotes the ratio of F to FG concentration.

### Chemical equations of blood coagulation

**Fig. 1:**
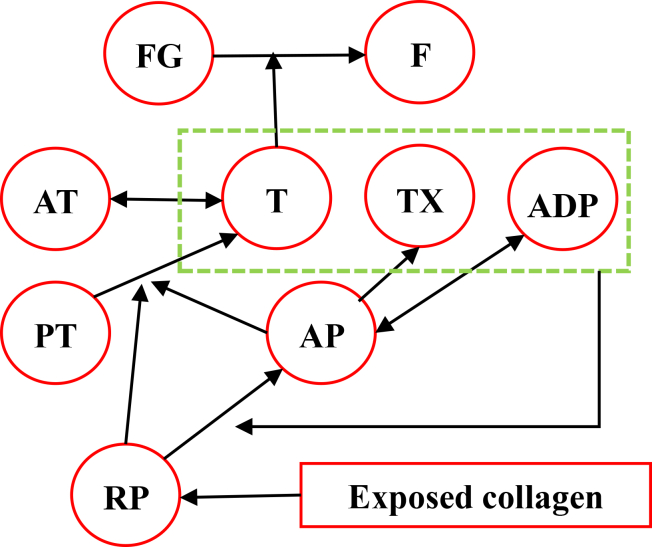
The cascade of chemical coagulation reactions begins with platelet exposure to collagen (at the base) and culminates in the formation of fibrin and fibrinogen (at the peak). This figure was created by the authors to illustrate the main steps of the modeled coagulation process.

All coagulation factors are assumed to be very small and move with the blood flow velocity, while only F and deposited platelets remain stationary and directly influence the flow field (53). In the present study, the CDR equation is employed to model the concentration of species. The general form of the CDR equation for each species is as follows (55):

(6) $$\frac{\partial C_{i}}{\partial t}+\left(\vec{V}.\nabla \right)C_{i}=D_{i}\nabla^{2}C_{i}+S_{i}\;i=1,2,\ldots ,9$$

where *C*_*i*_ denotes the concentration of a particular species, *D*_*i*_ represents the diffusion coefficient, and *S*_*i*_ corresponds to the production or consumption rate, as specified in Table 1. In this study, nine species are considered: RPs, APs, ADP, TX, PT, T, AT, FG, and F. The coagulation cascade is illustrated in Fig. 1.

**Table 1:** Production and consumption terms for each chemical reaction

Species	C_i_	C_i_ units	S_i_	Eq. no.	Ref.
Adenosine diphosphate (ADP)	C_ADP_	*μ*M	+*λ*_*ADP*_*k*_*RPA*_*C*_*RPV*_ – *k*_*ADP*_*C*_*ADP*_	(14)	Sorensen et al. (34)
Thromboxane (TX)	C_TX_	*μ*M	+*s*_*TX*_*C*_*AP*_ – *k*_*TX*_*C*_*TX*_	(15)	Sorensen et al. (34)
Prothrombin (PT)	C_PT_	*μ*M	–*βC*_*PT*_(*k*_*AP*,*T*_*C*_*AP*_ + *k*_*RP*,*T*_*C*_*RP*_)	(16)	Sorensen et al. (34)
Thrombin (T)	C_T_	U ml^–1^	–*ΓC*_*T*_ + *C*_*PT*_(*k*_*AP*,*T*_*C*_*AP*_ + *k*_*RP*,*T*_*C*_*RP*_)	(17)	Sorensen et al. (34)
Antithrombin (AT)	C_AT_	*μ*M	–*ΓβC*_*T*_	(18)	Sorensen et al. (34)
Fibrinogen (FG)	C_FG_	*μ*M	$-\frac{84C_{T}}{0.0072+C_{FG}}C_{FG}$	(19)	Govindarajan et al. (40)
Fibrin (F)	C_F_	*μ*M	$\frac{84C_{T}}{0.0072+C_{FG}}C_{FG}$	(20)	Govindarajan et al. (40)

When RPs encounter a damaged blood vessel (collagen layer), they become activated, releasing ADP, which subsequently activates additional platelets. Both APs and RPs adhere to collagen. The rate of platelet production or consumption is governed by the following relationships (34):

(7) $$S_{RP}=-k_{RPA}C_{RP}$$

(8) $$S_{AP}=+k_{RPA}C_{RP}$$

(9) $$M_{DP}=qK_{RPD}C_{RP}+qK_{APD}C_{AP}+K_{APA}C_{AP}\varphi$$

Here, *k*_*RPA*_ denotes the activation rate of the RP, *K*_*i*_ represents the heterogeneous reaction rate, *q* is the surface area not covered by platelets. The first term in Eqs. 7 and 8 represents the conversion of RPs to APs. The first two terms in Eq. 9 correspond to resting and AP deposition on the collagen layer, respectively, while the last term in Eq. 9 describes the adhesion of APs to the growing platelet plug. In this study, platelet-platelet adhesion is considered not only for the collagen layer but also for other upstream layers. Since deposited platelets adhere exclusively to the collagen layer or the platelet plug, the convection and diffusion terms for deposited platelets are omitted (38).

The ratio of deposited platelets to the maximum possible deposition (*φ*), the available surface area (*q*), and RP activation rate (*k*_*RPA*_) are determined by the following equations (34):

(10) $$\varphi \left(x,t\right)=\frac{\int_{0}^{t}M_{DP}dt}{M_{\max}}$$

(11) q(x,t)=1−φ

(12) $$k_{RPA}=\left\{ \;\begin{array}{c} 0,\Omega <1\\ \frac{\Omega }{t_{Act}},\Omega >1 \end{array}\right.$$

(13) $$\Omega =\frac{C_{ADP}}{C_{ADP,Cri}}+\frac{C_{TX}}{C_{TX,Cri}}+\frac{C_{T}}{C_{T,Cri}}$$

where *C*_*i*,*Cri*_ and *t*_*Act*_ represent the critical concentration of each species and the time constant for platelet activation, respectively. The other rates of production or consumption for the chemical reactions (Eqs. 14, 15, 16, 17, 18, 19, and 20) are provided in Table 1.

Inside the clot, F naturally exists in both polymeric and fibrous states, which significantly restricts its motion. Consequently, the convection term is omitted from the F concentration equation (40,44). *k*_*AP*,*T*_ and *k*_*RP*,*T*_ represent the rates of thrombin generation from PT on resting and APs, respectively. *β* is the unit converter from U ml^–1^ to *μ*M, while Γ represents the Griffith template model for the kinetics of heparin-catalyzed thrombin inactivation by AT (56), as described below:

(21) $$\Gamma =\frac{k_{T}C_{H}C_{AT}}{\alpha C_{AT,Dis}C_{T,Dis}+\alpha C_{AT}C_{T,Dis}+\alpha C_{AT,Dis}C_{T}+\alpha C_{AT}C_{T}}$$

### Geometry and boundary conditions

**Fig. 2:**
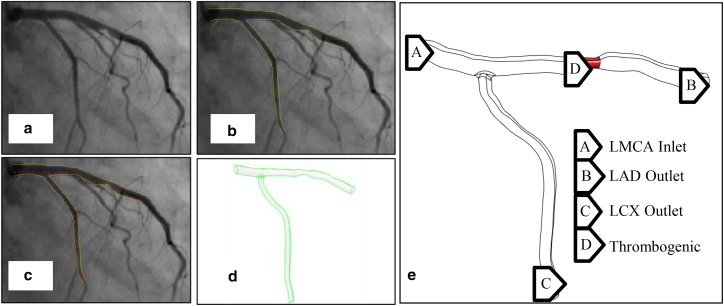
Steps to create 3D geometry for LMCA in patient no. 5. (*a*) Angiographic image of patient 5, (*b*) identification of the vessel wall boundary using green lines, (*c*) determination of vessel diameter, (*d*) creation of 3D geometry in CATIA software, and (*e*) identification of the inlet, outlet, and thrombogenic area.

The geometry utilized in this study is the left main coronary artery (LMCA) with the LAD-LCX (left anterior descending, left circumflex artery) branch, which was reconstructed using angiographic images from patients at the Tehran Heart Center (all imaging data are managed in accordance with ethical guidelines to ensure patient confidentiality and data protection, and all patient data are anonymized to safeguard privacy). Fig. 2 shows the steps to create 3D geometry of patient no. 5, as an example. Points on the angiographic images are marked using digitizing software (Fig. 2 *b*) to calculate the vessel diameter at each section. These measurements are then entered into CATIA software to generate a 3D model. This process was repeated for six different patients.

**Fig. 3:**
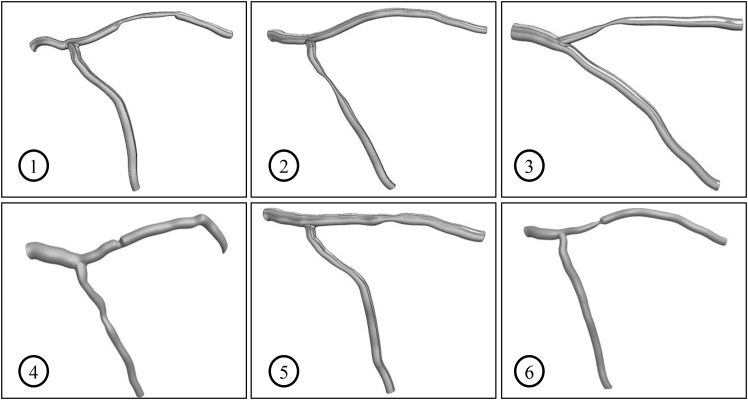
LMCA geometry of patients 1–6.

The geometry for patient no. 5 is reconstructed from angiographic images, revealing the following vessel characteristics: 44% stenosis in the LAD branch, stenosis length of 6 mm, and diameters of 4.8 mm for the LMCA, 3.4 mm for the LAD, and 2.9 mm for the LCX. According to (12), regions of the vessel wall that are exposed to shear stress greater than 7 Pa are at increased risk of thrombus formation. In this study, areas within the stenosis experiencing shear stress higher than 7 Pa are considered as thrombogenic areas, whereas previous studies (1,21,39) assumed the entire stenosed area as thrombogenic. The final coronary geometry for all patients is represented in Fig. 3, with the corresponding characteristics summarized in Table 2.

**Table 2:** Vessel characteristics of patients 1–6 (the percentage of stenosis is based on vessel diameter)

Patient no.	Diameter (mm)			Stenosed blood vessel		
	LMCR	LAD	LCX	Area	Percentage (%)	Length (mm)
1	4.10	3.50	3.00	LAD	63	20.45
2	4.40	2.90	2.81	LCX	71	22.70
3	4.70	3.30	3.10	LAD	75	28.40
4	5.00	3.20	3.38	LAD	28	3.00
5	4.80	4.30	2.90	LAD	44	6.00
6	4.10	2.90	2.70	LAD	65	17.00

To ensure the representativeness of patient-specific models, selection criteria included diversity in stenosis degree, location, and affected branches, with the aim of capturing a broad range of anatomical and pathological variations within the limitations of available data.

Given that the blood flow rate in the left coronary artery is from 0 to 4 × 10^–6^ m^3^/s (57) and considering that pulsatile flow does not affect the duration of channel occlusion, according to (58) the inlet blood flow is assumed to be constant at 2 × 10^–6^ m^3^/s (2 cc/s). However, due to the variation in clot size, which directly affects the hydrodynamic features, the equations are solved unsteadily. The species concentration at the inlet is assumed to follow the normal blood concentration. At the outlets, the pressure is assumed to be constant and the flux of all species is assumed to be zero. The vessel wall is assumed to be rigid in all cases, meaning that the blood flow velocity on the walls is zero. The species flux at the wall is assumed to be zero, except in the thrombogenic area, where the fluxes of the species, listed in Table 3, are assumed to be non-zero (1,21,34,38,39). Table 3 represents the boundary conditions for species with non-zero fluxes in the thrombogenic area.

**Table 3:** Boundary conditions for the species in the thrombogenic area

Species	Boundary condition
RP	*j*_*RP*_(*x*,*t*) = *qk*_*RPD*_*C*_*RP*_
AP	*j*_*AP*_(*x*,*t*)=(*qk*_*APD*_ + *φk*_*APA*_)*C*_*AP*_
ADP	*j*_*ADP*_(*x*,*t*) = –*λ*_*ADP*_*qk*_*RPD*_*C*_*RP*_
TX	*j*_*TX*_(*x*,*t*) = –*φs*_*TX*_
PT	*j*_*PT*_(*x*,*t*) = *βφk*_*AP*,*T*_*C*_*PT*_
T	*j*_*T*_(*x*,*t*) = –*φk*_*AP*,*T*_*C*_*PT*_

The coefficients for (1), (2), (3), (4), (5), (6), (7), (8), (9), (10), (11), (12), (13), (21) and Table 3 are represented in Table 4.

**Table 4:** The coefficient values used in the simulation (34)

Coefficient	Value	Coefficient	Value
*C*_*ADP*,*Cri*_	2 *μ*M	*k*_*AP*,*T*_	$3.69\times 10^{-9}\frac{U}{PLT.s.\mu M}$
*C*_*TX*,*Cri*_	0.6 *μ*M	*k*_*RP*,*T*_	$6.5\times 10^{-10}\frac{U}{PLT.s.\mu M}$
*C*_*T*,*Cri*_	$0.1\;\frac{U}{\text{ml}}$	*μ*	0.0035 Pa.s
*λ*_*ADP*_	$2.4\times 10^{-8}\frac{\text{nmol}}{\text{PLT}}$	*Ρ*	$1106.4\frac{\text{kg}}{m^{3}}$
*k*_*ADP*_ = *k*_*TX*_	$0.0161\frac{1}{s}$	*C*_*T*,*Dis*_	0.035 *μ*M
*k*_*T*_	$13.333\;\frac{1}{s}$	*C*_*AT*,*Dis*_	0.1 *μ*M
*s*_*TX*_	$9.5\times 10^{-12}\frac{\text{nmol}}{\text{PLT}.s}$	*α*	$\frac{1}{s}$
*β*	$9.11\times 10^{-3}\frac{\text{nmol}}{U}$	*t*_*Act*_	1 s
*M*_*max*_	$7\times 10^{6}\frac{\text{PLT}}{cm^{2}}$	*C*_*H*_	0.3 *μ*M
*K*_*RPD*_	$3.7\times 10^{-5}\frac{m}{s}$	*K*_*APD*_ = *K*_*APA*_	$4.6\times 10^{-5}\frac{m}{s}$
*D*_*AP*_ = *D*_*RP*_	$\left(13.61\dot{\gamma }+1.58\right)10^{-13}$	*D*_*ADP*_	$2.57\times 10^{-10}\frac{m^{2}}{s}$
*D*_*TX*_	$2.14\times 10^{-11}\frac{m^{2}}{s}$	*D*_*PT*_	$3.32\times 10^{-11}\frac{m^{2}}{s}$
*D*_*T*_	$4.16\times 10^{-11}\frac{m^{2}}{s}$	*D*_*AT*_	$3.49\times 10^{-11}\frac{m^{2}}{s}$
*D*_*FG*_	$1\times 10^{-12}\frac{m^{2}}{s}$	*r*_*F*_	55 nm

### Numerical implementation

**Fig. 4:**
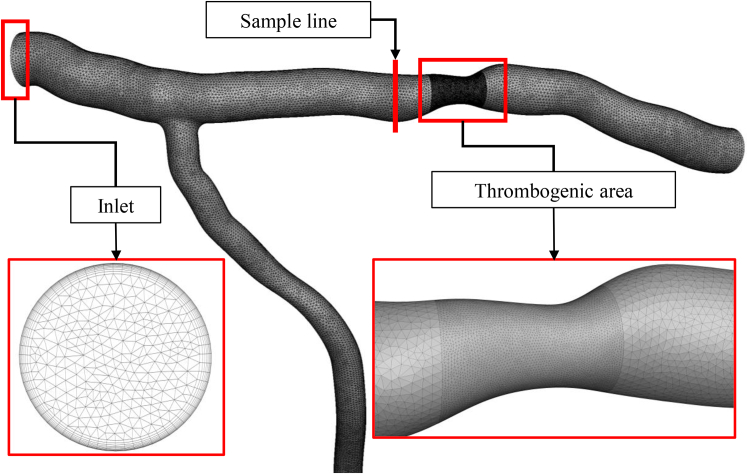
Computational grid with 1.681 million cells.

Numerical simulations are carried out using ANSYS FLUENT 2023 R2. The finite volume method is employed to solve the governing equations with the SIMPLE algorithm. To ensure numerical stability, all simulations were carried out using a fixed time step of 10^–3^ s, and second-order upwind discretization schemes were applied. The residuals for continuity, momentum, and species transport equations were set to 10^–4^. Throughout the unsteady simulations, the solutions remained smooth and convergent, and no numerical fluctuations in the flow field or species concentrations were observed. These observations confirm the numerical stability of the simulations under the selected solver settings. Fig. 4 illustrates the computational grid, comprising 1.681 million cells, which is used in the simulation of patient no 5.

**Fig. 5:**
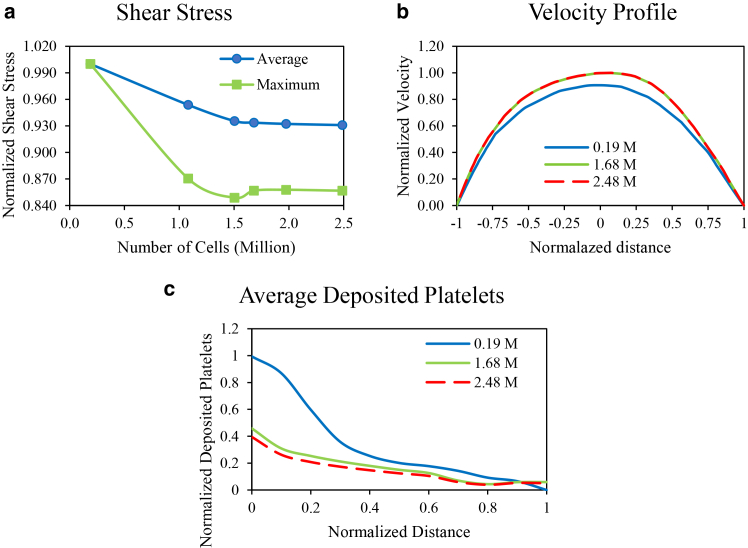
Computational grid study on (*a*) maximum local shear stress (*green*) and average local shear stress (*blue*) on the walls versus grid sizes, (*b*) velocity profile along a sample line (shown in Fig. 4) for grids with 0.188 million cells (*blue*), 1.681 million cells (*green*), and 2.488 million cells (*red*), (c) normalized deposited platelet in the thrombogenic area for grid size of 0.188 million cells (*blue*), 1.681 million cells (*green*), and 2.488 million cells (*red*).

To ensure that the results are independent of the grid size, a grid study is performed for the geometry of patient no. 5, as shown in Fig. 5, *a–c*.

Fig. 5 *a* illustrates the maximum and average local shear stress over entire walls as a function of the grid size. As shown in the figure, the results stabilize after 1.681 million cells, confirming that a mesh with 1.681 million cells is an appropriate choice. To further verify the grid suitability, grid independence is assessed for the velocity profile along the sample line (shown in Fig. 4) as well as for the normalized deposited platelet in the thrombogenic area (Fig. 5 *c*). The results are almost identical for 1.681 million and 2.488 million cells, indicating that a grid size of 1.681 million cells is optimal for this study.

## Results

### Validation

**Fig. 6:**
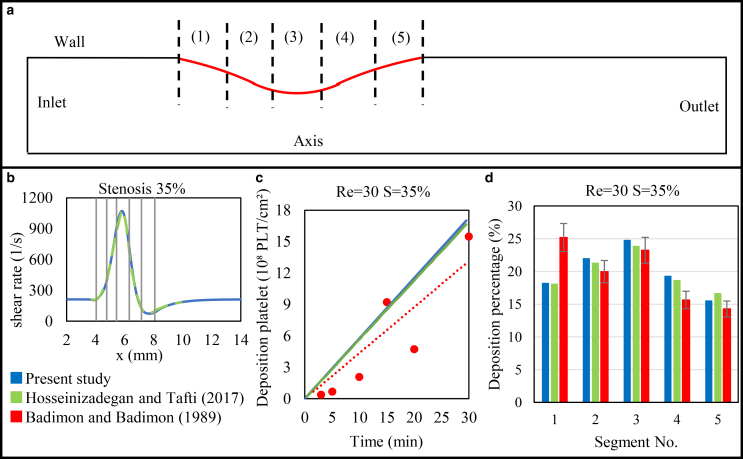
(*a*) Geometry of the validation case with a 35% stenosis. (*b*) Local wall shear rate along the stenosis, with gray lines indicating five equal thrombogenic regions. (*c*) Total deposited platelets over time. (*d*) Percentage of platelets deposited in each of the five stenotic regions. Blue, present study; green, Hosseinzadegan and Tafti (1); red, Badimón and Badimón (59).

For validation, we used the experimental conditions of Badimón and Badimón (59), in which whole blood was perfused through 2-mm aortic segments at flow rates of 10, 40, and 60 ml/min. These conditions correspond to Reynolds numbers of approximately 30, 120, and 180, computed using *Re* = *ρUD*/*μ* (*ρ* represents the density, *U* is the mean velocity, *D* = 2 mm is the vessel diameter, and *μ* refers to the dynamic viscosity of the blood). Fig. 6 *a* illustrates the geometry of the study. The stenosed region, which represents the thrombogenic area (*red line*), is divided into five equal segments after Hosseinzadegan and Tafti (1). Fig. 6, *b*, *c*, and *d* compare the current simulation results (*blue*), the numerical results (*green*), and the experimental data (*red*) calculated at 35% stenosis and the inlet Reynolds number of 30.

Fig. 6 *b* shows the local wall shear rate, with the gray lines marking the boundaries of the five thrombogenic areas. Initially, the shear rate remains constant for the first 4 mm. At the beginning of the stenosed region, the fluid velocity sharply increases due to the reduction in the cross-sectional area. This increase in velocity creates a gradient in the flow field, leading to an increase in the shear rate. The shear rate continues to rise, and reaches its peak at the apex of the stenosed region. Beyond this point, as the channel cross-sectional area increases, the fluid velocity decreases, causing a corresponding reduction in shear rate. In the fifth thrombogenic area, the formation of a vortex lowers the shear rate to less than its pre-stenosed value. Following this, the shear rate gradually increases, eventually returning to its initial state. The trends observed in this study are consistent with the results of Hosseinzadegan and Tafti (1), as well as experimental results of Badimón and Badimón (59).

Fig. 6 *c* illustrates the total deposited platelet over a 30-min period. As shown, the results align closely with both the simulation data and the experimental data, confirming the high accuracy of the model.

As previously mentioned, the stenosed region is divided into five sections, Fig. 6 *d* depicts the ratio of deposited platelet in each section relative to the total stenosed area. As shown in Fig. 6 *d*, the deposited platelets increase, reaching a maximum in the third section of the stenosis and decreasing thereafter. This trend is not solely due to the geometric narrowing but is strongly influenced by shear-dependent platelet transport. In our model, the effective platelet diffusivity follows the classical Keller relation (60), in which the diffusion coefficient increases linearly with the local shear rate ($D_{AP}=D_{RP}=\left(13.61\dot{\gamma }+1.58\right)10^{-13}$, Table 4). This shear-enhanced diffusivity substantially increases the near-wall platelet flux at the stenosis throat, despite the reduction in residence time. Consequently, the number of platelets reaching the surface per unit time increases in high shear stenosis regions, which explains the higher deposition. The results closely match those of Hosseinzadegan and Tafti (1). It is noteworthy that, in the third section of the stenosis, the results show excellent agreement with the experimental results of Badimón and Badimón (59). Additional comparison is carried out for different stenosis degrees (55% and 80%) and Reynolds numbers (120 and 180), further confirming the accuracy of the model.

**Fig. 7:**
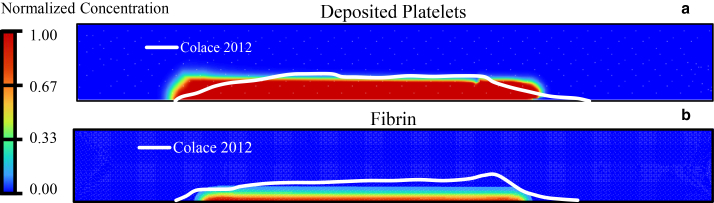
(*a* and *b*) Comparison of the present results with the experimental findings of Colace et al. (61) for deposited platelets (*a*) and fibrin (*b*)

To validate the dynamics of clot growth, the results of the present simulation are compared with experimental data provided by Colace et al. (61) and are shown in Fig. 7.

Fig. 7, *a* and *b*, shows a comparison between the dimensionless concentrations of deposited platelets and F obtained in the present study (concentration contours) and the experimental findings of Colace et al. (61) (*white line*). Comparison with experimental data by Colace et al. (61) shows that the main discrepancies appear near the upstream and downstream edges of the clot (clot shell), while agreement is strong in the central core region. This pattern reflects differences between the mechanisms governing the clot core, where growth is primarily driven by the main reactions (62) (platelet activation and deposition, ADP release, T generation, and F formation), which are explicitly represented in our nine species CDR model. In contrast, the clot shell is influenced by additional processes that are simplified or not included in our model (e.g., fibrinolysis, inhibitory pathways, and detailed spatial heterogeneity of the injury). In addition, the clot boundary is determined in the experiments from fluorescence-intensity thresholds (61), whereas in the simulations it is inferred from dimensionless concentration contours. These factors mainly influence the extent and shape of the clot envelope, leading to local deviations in the clot shell. Similar behavior has been observed in the studies of Govindarajan et al. (40) and Mitrophanov et al. (62). Nonetheless, since the clot height aligns with the experimental data, this suggests that the hydrodynamic modeling and chemical species representation are accurate.

### Hemodynamics

**Fig. 8:**
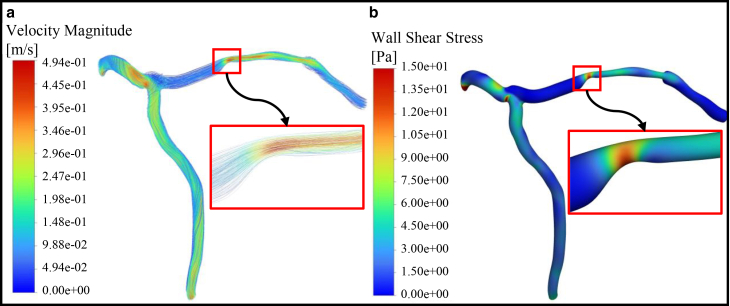
(*a*) Streamlines colored with velocity contours and (*b*) shear stress for patient no. 1.

This section focuses on the hemodynamic of blood flow within our geometries without considering chemical reactions. As previously mentioned, shear stress activates platelet receptors (13) and consequently leads to clot formation. In this study, hemodynamics is first analyzed to identify potential sites of clot formation. Fig. 8 illustrates the hemodynamic of blood flow, including velocity, streamlines, and shear stress in the LMCA of patient no. 1. As mentioned before, this patient has a 63% stenosis in the LAD branch.

Fig. 8 *a* shows the streamlines colored by velocity contour. In the stenosed region, the reduction in cross-sectional area leads to an increase in the flow velocity, which creates a strong velocity gradient on the vessel wall. This gradient results in high shear stress at the wall, as shown in the next part of the figure. Fig. 8 *b* depicts the shear stress distributions for patient no. 1. The highest shear stress values are observed in the stenosis (as shown in the *red area*).

**Fig. 9:**
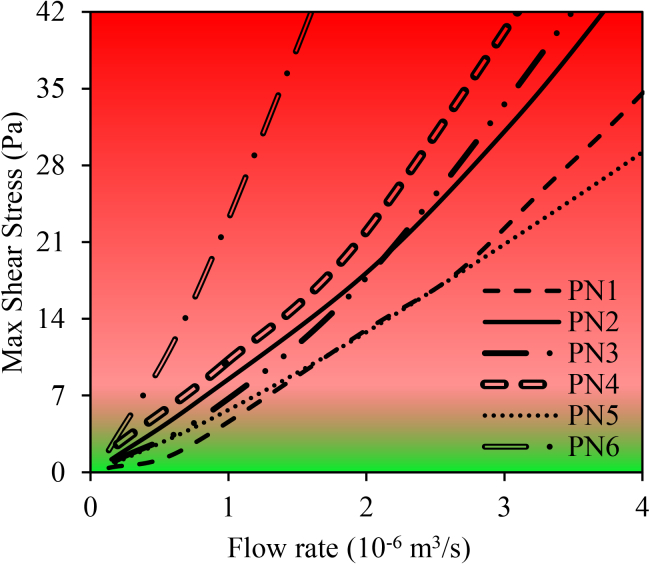
Maximum shear stress values versus flow rates across patients 1–6, with the green-shaded areas representing shear stress below 7 Pa and the red-shaded areas representing shear stress exceeding 7 Pa.

The maximum shear stress reaches 15 Pa across the stenosis, emphasizing the potential for thrombus formation in this region (12). As indicated in Malek et al. (12), regions of the vessel wall experiencing shear stress greater than 7 Pa are particularly prone to thrombus formation. To better illustrate these findings, Fig. 9 is prepared based on this threshold. This figure illustrates the maximum shear stress as a function of flow rate for patients 1–6. Green-shaded areas represent shear stress below 7 Pa (low-risk zone), while red-shaded areas denote shear stress above 7 Pa (high-risk zone).

**Fig. 10:**
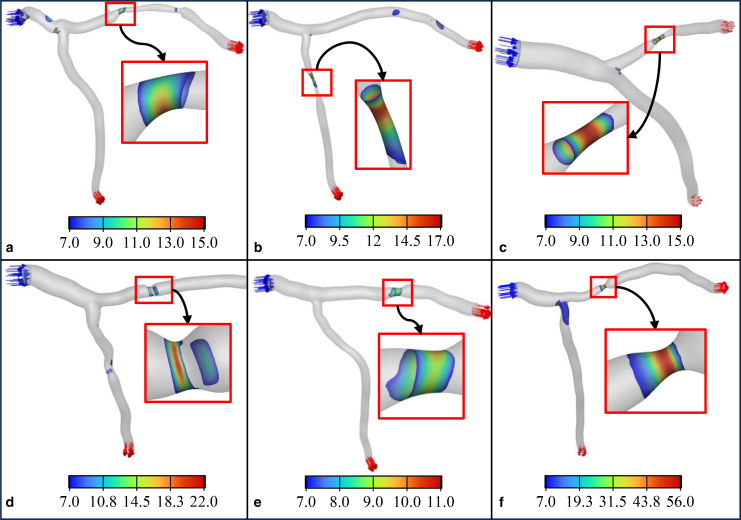
Distribution of regions with wall shear stress greater than 7 Pa in all six patient-specific geometries (panels *a*–*f*, corresponding to patients 1–6). Highlighted areas in the stenosed region indicate thrombogenic zones with elevated shear stress, which are potential sites for clot formation. These regions are mostly located within stenotic sections.

As shown in Fig. 9, patient no. 6 remains in the high-risk zone for almost all flow rates, while the other patients enter the high-risk zone at flow rates exceeding 1 × 10^–6^ m^3^/s. Furthermore, areas with shear stress greater than 7 Pa are identified and are represented in Fig. 10 for all patient geometries. Fig. 10 indicates shear stress greater than 7 Pa for all patients.

These high shear stress areas are mainly located within the stenosed zones in all patients. Consequently, regions with shear stress higher than 7 Pa in the stenosed zones are designated as thrombogenic areas. Although shear stresses above 7 Pa also occur at physiological sites such as bifurcation apex or sharp bends, these areas of intact, functional endothelial are preserved. In a healthy endothelium, elevated shear stress stimulates the production of nitric oxide and prostacyclin, two potent endogenous inhibitors of platelet activation and aggregation (63,64,65). This shear-mediated release of antithrombotic mediators prevents platelet adhesion despite the high shear environment. Therefore, high shear stress at bends or bifurcation apex does not promote thrombosis. In contrast, stenotic segments frequently exhibit endothelial dysfunction and reduce NO/PGI_2_ bioavailability, making shear stresses above 7 Pa pathologically thrombogenic only within the stenosis. The maximum shear stress has a different value for each patient according to the stenosis degree and vessel diameter. For example, for patients no. 3 and no. 6 (Fig. 10, *e* and *f*), the maximum shear stress is 15 and 56 Pa, respectively.

Fig. 10 *a* illustrates shear stress greater than 7 Pa for patient no. 1. At the onset of the stenosis, shear stress exceeds 7 Pa, increases to a maximum of 12 Pa, and then decreases. This pattern is consistent in other patients, as shown in Fig. 10, *b–f*, along the stenosis.

### Clot growth

**Fig. 11:**
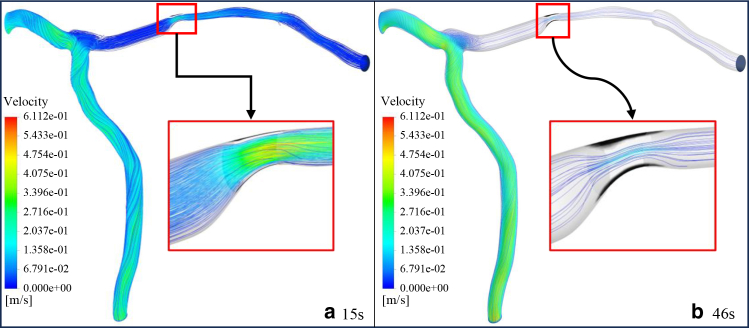
Clot growth and velocity streamlines for patient no. 1 at (*a*) 15 s and (*b*) 46 s. The black region indicates the growing thrombus, which increases resistance and redirects flow. Streamlines are colored by velocity magnitude, showing flow reduction in the thrombosed LAD branch and compensation via the LCX branch.

This section explores the dynamics of clot growth and its impact on the flow field. Fig. 11, *a* and *b* illustrates the streamlines, velocity contours, and clot growth at 15 s, when blood is still flowing through the LAD, and at 46 s, when increased hydraulic resistance reduces LAD flow to almost zero despite the lack of complete occlusion of the lumen, for patient no. 1.

Fig. 11 *a* illustrates the streamlines and clot growth for patient no. 1 after 15 s. At this situation, a thin layer of platelets (*black area*) has already covered the collagen surface. Fig. 11 *b* represents the streamlines and clot growth for the same patient after 46 s. As the clot grows (*black area*), the hydrodynamic resistance increases, causing the flow rate in the LAD branch to decrease until it reaches zero. After 46 s, the flow rate through the LAD branch has dropped to less than 1%, with almost no flow passing through this branch. In contrast, blood flow continues through the LCX branch. As the clot grows, the average blood flow velocity in the LCX branch increases from 22 to 28 cm/s, which is clearly observed by comparing the velocity contours in this branch at 15 and 46 s.

**Fig. 12:**
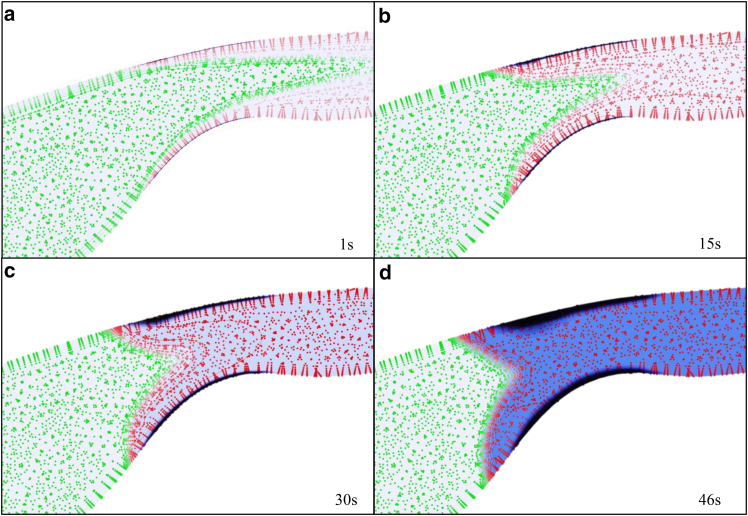
Time evolution of clot formation in patient no. 1 at four selected time points. Panels *a*–*d* correspond to 1, 15, 30, and 46 s, respectively. Green dots, resting platelets (RP); red dots, activated platelets (AP); blue background:, ADP concentration; black region, clot. The figure shows the progression from initial platelet deposition to significant clot growth and flow reduction

For better clarity in the clot formation process, the contours of AP, RP, and ADP, along with the clot area for patient no. 1, are plotted at several selected time points in Fig. 12. In this figure, the green dots represent RPs, the red dots represent active platelets, the blue area represents ADP, and the black area represents the clot. The green and red dots indicate the concentration of active and inactive platelets, respectively. They are visualized as discrete points solely for distinction from the clot region and the ADP enzyme and they do not imply a Lagrangian approach.

Although the LAD branch is not entirely occluded by the clot, the flow rate through it is reduced to less than 1% of the inlet flow rate. This phenomenon occurs due to the presence of the LCX branch. As the hydraulic resistance in the LAD increases, blood flow is diverted to another branch with less resistance. This indicates that changes in hydrodynamic resistance due to clot formation can alter the flow distribution in the vascular branches, significantly impacting overall blood circulation.

**Fig. 13:**
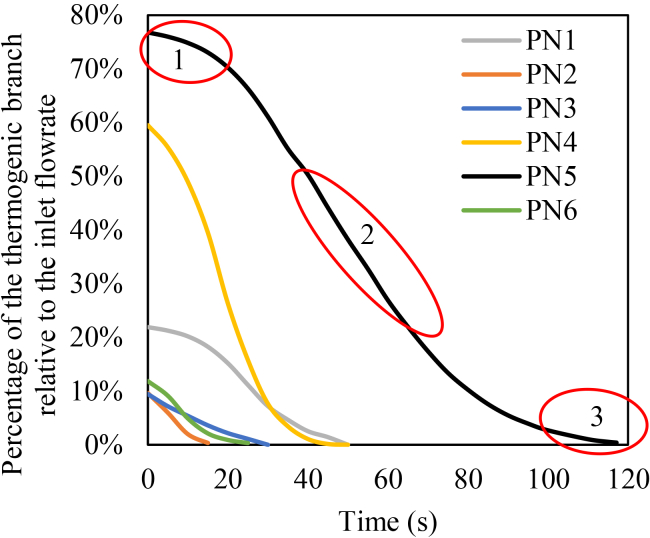
Percentage of relative thermogenic branch flow rate over time for patients 1–6. The three highlighted red regions indicate the progression of clot growth from initiation to complete flow occlusion.

Fig. 12 *a* illustrates the results after 1 s. Initially, there are few active platelets and most of the area is covered by RPs. At this stage, a thin layer of platelets adheres to the collagen surface but platelet activation is slow due to the low ADP concentration. As shown in Fig. 12 *b*, over time, the active platelets increase and the clot begins to grow (*black area*). Simultaneously, the secretion of ADP begins, as seen in Fig. 12 *c*, where the background changes to light blue, activating additional RPs. Finally, Fig. 12 *d* depicts a substantial increase in ADP concentration (*blue area*), which increases platelet activation and adhesion to the platelet plug, significantly promoting clot growth (*black area*). Based on these findings, Fig. 13 represents the percentage of blood flow through the thrombogenic branch over time for all patients.

As the clot grows, the hydrodynamic resistance of thrombogenic branch increases until its flow rate reaches zero. As shown in Fig. 13, the shortest time to reduce the flow rate to decrease to zero is observed for patient no. 2 at 15 s, while the longest time is observed for patient no. 5 at 117 s. The initial flow rate in the LAD branch of patient no. 2 is small due to the 73% stenosis (Table 2). As a result, a smaller clot is sufficient to occlude the branch. Consequently, the time required to reach complete occlusion is shorter depending on the degree of stenosis. The initial flow rate for patient no. 5 is large due to the 44% stenosis and large diameter. Therefore, the time required to reach complete occlusion is longer. The time required for the flow rate to reach zero depends on several factors, including the percentage of stenosis, branch diameter, location of the thrombogenic area, and its extent.

As illustrated in Fig. 13, the process of thrombus initiation and growth leading to complete flow cessation in the thrombogenic branch can be divided into three distinct phases. In the first phase, thrombus formation begins with the deposition of a thin layer of platelets at the thrombogenic area. In the second phase, ADP is activated, leading to the recruitment and activation of additional platelets, while the coagulation cascade is also triggered, further accelerating thrombus development. Finally, in the third phase, the growing thrombus increases the hydraulic resistance to such an extent that it overcomes the driving pressure of the flow. Consequently, the blood flow rate gradually decreases and eventually drops to zero, without complete occlusion of the branch by the thrombus.

To analyze the blood flow cessation time data, third-order regression is performed on the two features of vessel diameter and degree of stenosis. The regression results show that the stenosis degree has a stronger impact on the blood flow cessation time. According to these findings, the stenosis degree emerges as the dominant feature in predicting blood flow cessation time. Therefore, to achieve the most accurate and reliable predictions, the stenosis degree should be prioritized as a key factor in any prediction model aimed at prediction the blood flow cessation time.

Given the critical role of shear stress in thrombosis, accurate assessment of maximum shear stress in stenotic regions is crucial. Developing a clinical equation for rapid evaluation of stenosed arteries could significantly enhance diagnostic efficiency and improve patient management. In this study, a novel equation is proposed to estimate maximum shear stress in the left coronary artery of patients with stenosis. This equation, derived through numerical data fitting using the least-squares method with DataFit software, incorporates key hemodynamic parameters—including left coronary artery diameter, stenotic branch diameter, and degree of stenosis—and serves as a valuable tool for both clinical and computational applications.

(22) $$\tau_{\max}=4.99\times {\left({\dot{V}}_{\text{LMCA}}\right)}^{1.35}\times {\left(D_{\text{LMCA}}\right)}^{5.02}\times {\left(D_{\text{Ste}}\right)}^{-0.45}\times {\left({\left(\epsilon_{Ste}\right)}^{1.80}\left(1-\epsilon_{Ste}\right)\right)}^{3.56}$$

where ${\dot{V}}_{\text{LMCA}}$ is the LMCA flow rate, D_LMCA_ is the LMCA diameter, D_Ste_ is the diameter of the stenosed branch, and *ϵ*_*Ste*_ represents the percentage of stenosis. A maximum shear stress (τ_*max*_) greater than 7 Pa places the patient in the high-risk status, as such levels are associated with an increased likelihood of thrombus formation and coronary complications. Values below 7 Pa indicate a low-risk status. This index, using data obtained from patient angiography images, enables a rapid and accurate assessment of hemodynamic conditions and serves as a practical tool for clinical decision-making and optimal patient management.

$$\text{Patient}\;\text{status}=\left\{ \begin{array}{c} \text{high}-\text{risk},\tau_{\max}>7\\ \text{low}-\text{risk},\tau_{\max}<7 \end{array}\right.$$

**Fig. 14:**
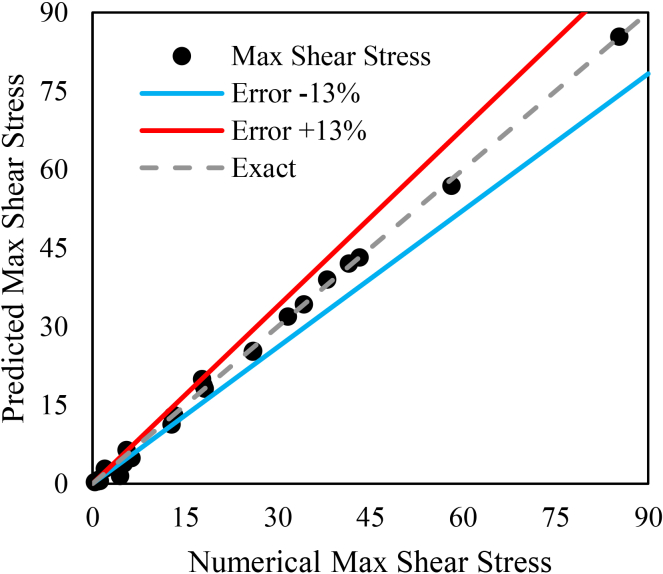
Maximum shear stress predicted from the Eq. 22 versus numerical results

Fig. 14 represents the maximum shear stress diagram derived from the proposed relationship (Eq. 22) along with the numerical results.

As shown in Fig. 14, the proposed equation predicts maximum shear stress values exceeding 7 Pa with an error margin of less than 13%.

## Discussion

Clot formation is strongly influenced by shear stress. In regions of the vessel wall where shear stress is greater than 7 Pa, the probability of clot formation significantly increases (12). Fig. 8 *a* illustrates the streamlines colored with velocity contours for patient no. 1. In the stenotic region, the reduction in cross-sectional area caused accelerates the blood flow along the occlusion and creates a high velocity gradient. This results in high shear stress, as clearly depicted in Fig. 8 *b*. Fig. 9 represents the maximum local shear stress as a function of flow rate for all six patients. Shear stress at the stenosis apex increases due to decrease in cross-sectional area, creating gradients that trigger platelet activation. It is evident that all patients fall within the range associated with a high probability of clot formation. Furthermore, based on Fig. 10, regions with shear stress greater than 7 Pa are identified as thrombogenic areas.

Fig. 12 illustrates the progress of clot formation, showing the distribution of active platelets, inactive platelets, and ADP at 1 and 46 s. As the thrombogenic area develops in the stenotic region, RPs collide with the exposed wound surface (collagen layer), resulting in the deposition of a thin platelet layer. The inactive platelets are subsequently converted to active platelets, leading to the secretion of ADP, which further activates surrounding inactive platelets. Consequently, the number of active platelets increases, and these active platelets adhere to the previously formed platelet plugs, as described by Eq. 9. This process causes the gradual growth of the clot. Clot growth narrows the vessel, thereby increasing the flow velocity and raising the hydraulic resistance, redirecting the flow toward another branch with less resistance. It is worth noting that the resistance inside a pipe is directly related to its average flow velocity.

Fig. 11 illustrates the streamlines along with the clot growth for patient no. 1. By 46 s, the flow rate through the LAD branch drops to zero, despite the fact that the vessel, unlike the single-branch vessel studies (53), is not completely occluded by clot. As the clot grows, the pressure loss increases and, according to Eq. 3, this increase in resistance gradually redirects the flow, causing the flow rate through the LCX branch to increase until the entire inflow is directed through the LCX branch. Thus, the presence of the second branch causes the flow rate through the stenosed branch to reach zero with a smaller increase in hydraulic resistance compared with single-branch studies (39), eliminating the need for complete stenosis for the flow rate to drop to zero. Bifurcations enable flow to be diverted, so complete occlusion is not required to achieve zero flow in one branch; resistance alone is enough. This situation poses a greater risk because a sudden increase in flow rate within the LAD branch, potentially induced by elevated hydraulic resistance in the LCX branch (e.g., due to lipid plaque formation), can lead to destabilization and embolization of a clot, ultimately increasing the likelihood of stroke.

Fig. 13 depicts the percentage of flow through the thrombogenic branch over time. Patient no. 5 exhibits the longest times to reach zero flow, while patients no. 2 and 3 show the shortest times. According to Table 2, the diameter of the LAD branch and the stenosis degree are 4.3 mm and 44% for patient no. 5. The large diameter in patient no. 5 results in initially higher flow rates and prolongs the time required for clot-induced hydraulic resistance to halt the flow. The cross-sectional area in the stenosis region for patient no. 5 is 8.13 mm^2^. This relatively large area contributes to a significant delay in the cessation of blood flow. For patient no. 3, the LAD diameter and stenosis percentage are 3.3 mm and 75%, respectively. For patient no. 2, who has a stenosis in the LCX branch, the LCX diameter and stenosis percentage are 2.9 mm and 71%, respectively. The high stenosis percentages in these patients results in rapid flow cessation. Additionally, the smaller cross-sectional area in the stenosis for patient no. 2 causes the flow rate to drop to zero faster than in patient no. 3. Smaller diameters and higher stenosis degrees accelerate the flow resistance buildup, leading to faster flow cessation compared with wider or less-stenosed branches.

According to Fig. 13, as the thrombogenic branch flow rate approaches zero, the rate of flow reduction slows. This phenomenon occurs because a lower flow rate reduces platelet adhesion to the platelet plug. A similar pattern is observed during the early stages of clot growth under high-flow conditions. In these cases, the rapid blood flow limits the opportunity for platelet deposition, as platelets have less time to adhere.

The empirical relationship proposed in Eq. 22 allows for the estimation of maximum shear stress using simple anatomical and flow parameters, such as vessel diameter and stenosis severity. Since high shear stress is a key factor in clot formation, this equation can help identify patients at thrombotic risk even when stenosis is not geometrically severe. It may assist physicians in early diagnosis of high shear stress and preventive decision-making.

## Conclusion

This study investigates clot formation in the left coronary bifurcation of patients with stenosis. The analysis focuses on vessel wall shear stress and identified high-risk areas. In addition, the effects of vessel diameter and stenosis percentage on maximum shear stress, clot growth, and time to zero flow in the stenosed branch are evaluated. The key findings of this study are summarized as follows.

• In our cases, the stenosed regions definitely experience shear stress exceeding 7 Pa at 2 × 10^-6^ m^3^/s (2 cc/s) flow rates.
• As expected, the highest shear stress is observed along the stenosis regions, significantly increasing the probability of clot formation and growth in this area.
• The blood flow cessation time in the stenosed branch is affected by the branch diameter, the degree of stenosis, and the cross-sectional area at the stenosis site. Among these factors, the stenosis degree has the most pronounced effect on flow cessation.
• Higher stenosis percentages and smaller branch diameters lead to shorter times for flow cessation. For the same stenosis percentage, smaller cross-sectional areas result in faster flow cessation.
• In bifurcated vessels, complete occlusion by thrombus is not necessary for blood flow cessation; even minor clot growth and subsequent increase in hydraulic resistance can significantly reduce flow rate to near zero.
• Maximum shear stress is inversely proportional to the diameter and directly proportional to the stenosis percentage. These two parameters are the most critical factors influencing maximum shear stress.

### Future work

To further improve the physiological relevance and clinical applicability of the model, future work may include these suggestions.

• Implementation of more realistic boundary conditions, such as Windkessel models along with pulsatile inflow, to better represent downstream vascular resistance and time-dependent pressure-flow behavior.
• Investigating pharmacological strategies that target key biochemical factors involved in thrombus formation to delay flow occlusion is a promising research direction.
• Exploring the effects of pathological conditions, such as cardiac arrhythmias, on thrombus initiation and growth may provide valuable insights into patient-specific thrombosis risk and mechanisms of flow blockage.
• Given the limited number of patient-specific models, inter-patient comparisons in this study are primarily qualitative. Future larger cohort studies are needed to enable rigorous statistical analyses.

## Data and code availability

• Patient-specific angiographic images used in this study cannot be placed in a public repository due to privacy and ethical restrictions.
• Anonymized derived data, including reconstructed geometries and simulation results, are available from the corresponding author upon request.

## Acknowledgments

The authors thank the Tehran Heart Center for providing the anonymized angiographic data used in this study.

## Author contributions

Writing – original draft, M.R.; writing – review & editing, M.R. and B.F.; visualization, M.R.; methodology, M.R. and B.F.; investigation, M.R. and B.F.; conceptualization, M.R. and B.F.; data curation, M.R.; formal analysis, M.R.; resources, M.R.; software, M.R.; resources, M.R.; validation, M.R.; supervision, B.F.

## Declaration of interests

The authors declare no competing interests.
